# Friedelin Attenuates Neuronal Dysfunction and Memory Impairment by Inhibition of the Activated JNK/NF-κB Signalling Pathway in Scopolamine-Induced Mice Model of Neurodegeneration

**DOI:** 10.3390/molecules27144513

**Published:** 2022-07-14

**Authors:** Marva Sandhu, Hafiz Muhammad Irfan, Shahid Ali Shah, Madiha Ahmed, Iffat Naz, Muhammad Akram, Humaira Fatima, Ayesha Shuja Farooq

**Affiliations:** 1 College of Pharmacy, University of Sargodha, Sargodha 40100, Pakistan; marvasandhu90@hotmail.com (M.S.); muhammad.akram@uos.edu.pk (M.A.); 2 Drugs Control and Traditional Medicines Division, NIH, Islamabad 45500, Pakistan; 3 Department of Biology, The University of Haripur, Haripur 22620, Pakistan; shahid.ali@uoh.edu.pk; 4 Neuro Molecular Medicines Research Center (NMMRC), Peshawar 25000, Pakistan; 5 Shifa College of Pharmaceutical Sciences, Shifa Tameer-e-Millat University, Islamabad 44000, Pakistan; madiha.scps@stmu.edu.pk; 6 Department of Biology, Science Unit, Deanship of Educational Services, Qassim University, Buraidah 51452, Saudi Arabia; iffatkhattak@yahoo.com; 7 Department of Pharmacy, Faculty of Biological Sciences, Quaid-i-Azam University, Islamabad 45320, Pakistan; humaira.fatima23@gmail.com or; 8 Department of Biochemistry, Science Unit, Deanship of Educational Services, Qassim University, Buraidah 51452, Saudi Arabia

**Keywords:** scopolamine, p-JNK, friedelin, neurodegeneration, Alzheimer’s disease, oxidative stress, swiss albino mice

## Abstract

Oxidative stress (OS) and c-Jun N-terminal kinase (JNK) are both key indicators implicated in neuro-inflammatory signalling pathways and their respective neurodegenerative diseases. Drugs targeting these factors can be considered as suitable candidates for treatment of neuronal dysfunction and memory impairment. The present study encompasses beneficial effects of a naturally occurring triterpenoid, friedelin, against scopolamine-induced oxidative stress and neurodegenerative pathologies in mice models. The treated animals were subjected to behavioural tests i.e., Y-maze and Morris water maze (MWM) for memory dysfunction. The underlying mechanism was determined via western blotting, antioxidant enzymes and lipid profile analyses. Molecular docking studies were carried out to predict the binding modes of friedelin in the binding pocket of p-JNK protein. The results reveal that scopolamine caused oxidative stress by (1) inhibiting catalase (CAT), peroxidase enzyme (POD), superoxide dismutase (SOD), and reduced glutathione enzyme (GSH); (2) the up-regulation of thiobarbituric acid reactive substances (TBARS) in mice brain; and (3) affecting the neuronal synapse (both pre- and post-synapse) followed by associated memory dysfunction. In contrast, friedelin administration not only abolished scopolamine-induced oxidative stress, glial cell activation, and neuro-inflammation but also inhibited p-JNK and NF-κB and their downstream signaling molecules. Moreover, friedelin administration improved neuronal synapse and reversed scopolamine-induced memory impairment accompanied by the inhibition of β-secretase enzyme (BACE-1) to halt amyloidogenic pathways of amyloid-β production. In summary, all of the results show that friedelin is a potent naturally isolated neuro-therapeutic agent to reverse scopolamine-induced neuropathology, which is characteristic of Alzheimer’s disease.

## 1. Introduction

Neurodegenerative disorders (ND) are characterized by impaired structures and functions of neurons or neuronal cells. Parkinson’s disease, multiple sclerosis, Huntington’s disease and Alzheimer’s disease (AD), along with associated dementia, are major examples of ND [1]. AD being the leading cause of memory impairment and dementia among geriatrics has the most devastating effect on cognitive skills leading to emotional distress. The etiological characterization of AD has yet to be completely explicated, however, the neuropathological effects of amyloid-β (Aβ) aggregates and neurofibrillary tangles (NFTs) owing to hyperphosphorylated tau proteins have been identified [2]. The Aβ aggregates are deposited on extracellular surfaces of the neuronal cells due to the activation of three mitogen activating protein kinases (MAPK) pathways in neurons i.e., c-Jun N-terminal kinase (JNK), p38, extracellular signal-regulated protein (ERK) and nuclear factor- κB (NF-κB) ultimately leads to neurodegeneration [3]. Specifically, the up- regulation of NF-κB activity not only exacerbates Aβ production and deposition, but also enhances pro-inflammatory cytokines and chemokine’s expression inducing AD neurotoxicity [4]. NF-κB is found to be overexpressed in degenerated cholinergic neurons and the proximal glial nuclei of AD affected brains [5]. Scopolamine-induced neurodegenerative models are widely used for identification of neuroprotective agents, as it is associated with enhanced acetyl cholinesterase (AChE) activity, increased reactive oxygen species (ROS) levels in brain, increased the levels of inflammatory mediators such as COX-II in brain, and increased levels of thiobarbituric acid reactive substances (TBARS) [6]. With regard to AD, scopolamine induces reversible symptoms that mimic dementia by deregulating cholinergic function and promoting Aβ deposition, both being the hallmarks of the said disease [7]. As of today there are few FDA-approved drugs in the market for treatment, and several researchers have dedicated their research/studies on plant-derived or isolated compounds as a potential source of either novel or more effective neuro-therapeutic agents [8]. Pentacyclic triterpenes are the most abundantly found and versatile group of naturally occurring compounds. Both the anti-inflammatory and anti-oxidant activities of various pentacyclic triterpenes such as oleanolic acid, ursolic acid, maslinic acid, corosolic acid, erythrodiol and glycyrrhizic acid have already been established in murine models [9,10]. Therefore, in the current study, the aforementioned attributes of friedelin, a pentacyclic triterpenoid, were sought against neuropathologies specifically associated with AD.

**Figure 1:**
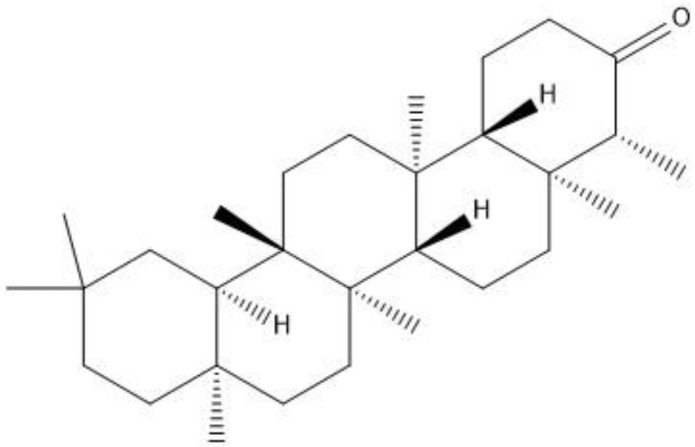
2D structure of friedelin.

Friedelin belongs to class of pentacyclic triterpenes (Figure 1) abundantly found in several species of plants. Friedelin is reported to possess anti-cancer [11], anti-ulcerogenic [12], anti-inflammatory, antipyretic, analgesic [13], in vitro antioxidant [14], hepatoprotective, anti-hyperlipidemic [15], and neuroprotective properties by hindering Aβ aggregation in in vitro models [16]. Owing to the necessity for the testing of this compound in in vivo models, it creates the baseline for the current investigation, in which the effects of friedelin was evaluated in an animal model of scopolamine-induced oxidative stress mediated phosphorylated JNK activation, memory impairment, neurodegeneration, neuroinflammation and synaptic dysfunction were appraised. This may be attributed to its antioxidant/anti-neuroinflammatory/anti-neuroapoptotic effects through the inhibition of phosphorylated JNK protein. 

## 2. Materials and Methods

*Molecular docking methodology:* Molecular docking studies were performed using AutoDock Vina in the PyRx 0.8 [17], to predict binding modes of friedelin in the binding pockets of JNK protein retrieved from the Protein Data Bank (PDB) having ID: 3V6S (chainA). The structure of Friedelin was created with ChemSketch and converted into PDB format using the Discovery Studio Visualizer (DSV), which was then transformed to Protein Data Bank, Partial Charge, and Atom Type format (PDBQT) for advance molecular docking evaluation. The 3D structure of the target protein, i.e., JNK, was downloaded from the PDB (PDB ID 3v6s) with a resolution 2.97 Å and R-Value 0.263 (Free), 0.216 (Work), 0.219 (Observed). The 3D structure of the target protein, JNK, was obtained experimentally by the X-ray diffraction method [18]. The downloaded protein was opened in DSV, and the H_2_O molecules and chain B were removed. Protein 3D protonation was carried out followed by the minimization of energy for the stability of the target protein by means of the default PyRx parameters. Default settings of AutoDock Vina were used for the docking studies. Twenty conformations were formed and top ranked conformation was selected on the bases of the docking score for additional analysis.

*Chemicals:* Scopolamine, phosphate buffer saline tablets (PBS), sodium dodecyl sulphate (SDS), ammonium per sulphate (APS), acrylamide, bis-acrylamide, trizma base, potassium chloride (KCl), and sodium chloride (NaCl) were procured from Sigma Aldrich Chemical Co., St. Louis, MO, USA and monoclonal antibodies were purchased from Daejung Chemicals & Metals Co. Ltd., Siheung-si, Korea. The isolated test compound (friedelin) was provided by the courtesy of Natural Product Research Lab, Department of Pharmacy, Quaid-i-Azam University, Islamabad, Pakistan. Prior work has been carried out for the isolation of the subject compound from *Quercus dilatata* L., based upon the existing indigenous knowledge of medicinal uses of this plant specie. Friedelin was isolated from the n-hexane fraction of crude methanol extract of aerial parts of *Quercus dilatata* by bioassay guided separation techniques [19]. The purity of the compound was confirmed by HPLC-DAD analysis (15 mg, purity > 99%) carried out at the Natural Product Research Lab, Department of Pharmacy, Quaid-I-Azam University, Islamabad, Pakistan. Briefly, the RP-HPLC based purification of friedelin was checked on a Shimadzu HPLC system comprised of a SCL-10AVP controller, a DGU-14A degasser, a FCV-10ALVP low pressure mixer, an LC-10AT VP pump coupled with 3D-PDA detector (SPD-M10A VP), and LC solution software. The column used was an Agilent Zorbax C8 (5µm; 4.6 mm × 250 mm). The elution was done isocraticaly using the mobile phase which was composed of methanol and distilled water (9:1). The flow rate was 1 mL/min and the injection volume was 50 µL. The chromatogram was obtained at 207 nm (at λ_max_). The retention time of the friedelin standard and sample was 4.1 min with a total run time of 10 min. The concentration of the standard and sample was 100 µg/mL and 400 µg/mL, respectively. The concentration of the sample was kept fourfold higher than the standard to detect the impurities. The standard and sample were compared using retention time, a 3D-graph and UV-spectrum. The comparative chromatograms and 3D graph are given in Table 1. The original chromatograms with additional parameters have also been given in the mentioned table.

*Mice grouping*: To conduct these experiments, adult male Swiss albino mice were purchased from the NIH (National institute of Health) Veterinary sub division, and were brought to the Neuro Molecular Medicines Research Centre, (NMMRC), Peshawar. The number of animals in each group were calculated using the resource equation approach [20] and keeping the 3Rs (replacement, reduction and refinement) guidelines [21] for the use of animals in research in consideration. The experimental mice were placed in labelled Biobase cages with free access to food and water, and were allowed to acclimatize to the environment. The animal study protocol was approved by the Institutional Review Board (or Ethics Committee) of the University of Sargodha (protocol code SU/ORIC/2862 and date of approval 23 September 2021).

The male mice of 30–32 g mean body weight were placed in separate cages with a controlled environment, i.e., light/dark cycle (12/12 h), 25 ± 01 °C temperature and ad libitum supply of clean water and standardized pellet food. As per animal ethics committee recommendations, all of the research animals were handled with care and empathy. The doses of scopolamine and friedelin were selected and optimized from the literature review [22]. Friedelin isolated from different plants had already been reported to have significant in vivo activities in animal models of hepatotoxicity [15], gastro-protective activities [12], and anti-inflammatory and antipyretic activities [23]. The mice were later randomly distributed into four groups (*n* = 5), C = control group (vehicle control group), S = scopolamine treated group (1 mg/kg), S + F = scopolamine (1 mg/kg) + friedelin (30 mg/kg) and F = friedelin treated group (30 mg/kg).

*Drug administration and experimental design*: All experimental drugs were dissolved in normal saline and were dispensed interaperitonealy (i.p.). The animals were caught by tail with a hand wrapped around the neck region in a way to expose their ventral side upwards. The drug was administered in the left lower quadrant of the abdominal cavity with caution to avoid internal organ damage. Scopolamine was injected i.p. for 21 days, while the test drug, friedelin, was administered every day for the last 14 days. At the 10th day from the start of drug induction the training of mice (for behavioural tests) was started, and this continued for three days; after a rest period of two days the mice were subjected to the final tests for five consecutive days after 30 min of the drug’s administration. After one day of rest, the final probe test was carried out and post-test the mice were subjected to anaesthesia for blood collection through cardiac puncture, and afterwards brains were collected.

### 2.1. Behavioural Tests

To demonstrate the therapeutic efficacy of friedelin on scopolamine-mediated memory dysfunction, two well-known behavioural tests were executed. Scopolamine was injected interaperitonealy (i.p.) to the mice of group II and III without and with friedelin, respectively. Later, all mice were tagged and randomly divided into four (04) groups, while the behavioural studies were carried out as a single blind trial. The researcher carrying out the behavioural tests was kept blind of the tags and the mice treatment group.

*Morris Water Maze (MWM) Test:* To probe the hippocampus dependent long-term spatial learning capabilities of mice, the MWM behavioural task was performed. The design and dimensions of the MWM test apparatus were according to the previously reported details [24]. Before the actual commencement of the tests, mice were trained for swimming (two times/day) to acclimatize them with the water tank and platform for three days. Later, the escape latency for each mouse was calculated for a period of 60 s to find the hidden/submerged platform, and this practice continued for five days. If mouse failed to find the platform in given time (60 s) it was manually directed and placed on a platform for at least 10 s. The escape latency time (sec) for each day was recorded and maintained. Mice were placed on rest for a period of two days before final probe testing, i.e., the platform was hidden and the mean time spent by each mouse in the target quadrant was calculated.

*Y-Maze test:* The Y-maze behavioural task was performed as executed earlier [25]. The Y-maze apparatus consists of three arms with dimensions of 50 × 10 × 20 cm^3^ (L × W × H) connected at an angle of 120° to each other. The mice were acclimatized to this new environment for 10 min each time. Afterwards, each mouse was placed in the middle of the maze one by one and was allowed to explore the maze spontaneously in all three arms for 8 min. Total arm entries of each mouse and successive triplet entries were computed employing software and the percentage of alternations was calculated using the following formula:

$$Percentage\;alternations\;\left(\%\right)=\frac{Number\;of\;successive\;triplets\;}{Total\;arm\;entries-2\;}\times 100$$

The spatial memory function of mice was correlated positively with the percentage of alternations.

### 2.2. Western Blotting Analysis

All of the mice were euthanized after being given anesthesia by mild chloroform inhalation at the end of the 21st day of treatment, and behavioral studies mentioned in above sections. The mice were decapitated and the hippocampal part of the brain was carefully extracted, then quickly transferred to RNAlater (Sigma Ald. R0901) solution and PBS (1:1) and placed on ice. The hippocampal tissue was homogenized in total protein extraction reagent (T-PER by Thermo Scientific 1632086, Waltham, MA, USA) solution, and the tissue supernatant was collected and stored at −20 °C for future analysis. The total protein concentration was quantified employing a Bio-Rad protein estimation assay kit (5000001EDU) and absorbance was taken at 595 nm. All of the protein samples were normalized to 30 µg/group, and gel electrophoresis was performed using SDS-PAGE (12–15%). Running conditions were maintained at 50 mA for the first 20–30 min and then switched to 120 V for almost 1–1.5 h until the run was complete. Proteins from gel were trans-blotted to a PVDF membrane using the semi-dry transblott technique. Several mouse derived monoclonal primary antibodies procured from Santa Cruz Biotech Inc. (Dallas, TX, USA), such as anti-Iba (sc-32725), anti-GFAP (sc-33673), anti-p-JNK (sc-6254), anti-caspase-3 (sc-7272), anti-NF-κB (sc-8008), anti-PARP-1, anti-COX-II (sc-376861), anti-β-actin (sc-47778), anti-Aβ (sc-28365), anti-SYP (sc-17750), anti-PSD95 (sc-71933), anti-BACE1 (sc-337) and antibodies of dilution 1:1000 in PBS were applied followed by the application of 1:2000 anti-mouse HRP conjugated secondary antibody (0000375517). The X-ray films were developed and analyzed using ImageJ software [26].

### 2.3. Biochemical Analysis of Plasma

Upon the completion of drug treatment, the mice were euthanized and the collected blood was used for biochemical analysis of total cholesterol, high density lipoproteins (HDL), low density lipoproteins (LDL), very low density lipoproteins (VLDL), and triglycerides levels (TGL).

### 2.4. Antioxidant Enzyme Profiling of Brain Homogenates

*Catalase assay (CAT):* Catalase activity (CAT) was assessed through the method developed earlier with a few modifications [27]. The 3 mL of reaction mixture consisted of 2500 µL phosphate buffer (50 mM, pH 5.0), 400 µL of H_2_O_2_ (5.9 mM) and 100 µL of brain homogenate supernatant. Variation in absorbance of the reaction mixture was taken at 1 min intervals at 240 nm. The change in absorbance of 0.01 units/min was considered as one unit of activity.

*Peroxidase assay (POD):* The previously reported method with slight modifications was employed to measure the peroxidase activity [27]. The reaction mixture for peroxidase assay consists of 2500 µL phosphate buffer (50 mM) at pH 5.0, 300 µL H_2_O_2_ (40 mM), 100 µL of guaiacol (20 mM) and 1000 µL supernatant of brain homogenate. The variation in reaction mixture absorbance was taken at one min intervals at 470 nm. One unit of POD activity was regarded as a change in absorbance of 0.01 units/min.

*Superoxide dismutase assay (SOD):* This assay was performed with a slight modification, as reported earlier [27]. To estimate the superoxide dismutase (SOD) activity reaction mixture consists of 100 µL phenazine methosulphate (186 µM), 1200 µL sodium pyrophosphate buffer (0.052 mM, pH 7.0) and 300 µL brain homogenate supernatant. To initiate enzymatic activity, 200 µL of NADH (780 µM) was added to the reaction mixture, and then after 1 min, 1000 µL of the stopping agent, glacial acetic acid, was added. The absorbance of reaction mixture at 560 nm was measured to determine the amount of chromogen formed, and results were expressed as units/mg of protein.

*Reduced glutathione assay (GSH):* For estimation of the reduced glutathione levels, the proteins were precipitated in 1000 µL of brain homogenate mixed with equal parts of 4% sulfosalicylic acid solution according to the method previously reported [28]. The reaction mixture was incubated at 4 °C for 1 h, then centrifuged for 20 min at 4 °C and 1200× *g*. The reaction mixture contained 2700 µL 0.1 M phosphate buffer of pH 7.4, 100 µL centrifuged aliquot and 200 µL (100 mM) DTNB solution. The immediate absorbance of the mixture was recorded at 412 nm. The reduced glutathione levels were stated as µM/g brain tissue.

*Estimation of lipid peroxidation (TBARS):* A lipid peroxidation (TBARS) assay was carried out after slight modifications in the method developed earlier [29]. In the current assay, 1000 µL of the reaction mixture was comprised of 580 µL of 0.1 M phosphate buffer (pH 7.4), 200 µL 100 mM ascorbic acid, 200 µL brain homogenate supernatant, and 20 µL 100 mM ferric chloride. The final mixture was incubated for 1 h in a shaking water bath assembly maintained at 37 °C. 1000 µL of 10% trichloroacetic acid (TCA) solution was added as a stopping agent. Next, 1000 µL of 0.67% thiobarbituric acid (TBA) was added to the reaction tubes and these were placed in a boiling water bath for 20 min and then quickly shifted to a crushed ice bath and later centrifuged for 10 min at 2500× *g*. The amount of lipid peroxidation (TBARS) formed in all of the samples were quantified by taking the spectrophotometric absorbance of the supernatant at 535 nm. The results were expressed in units nM TBARS/min/mg of brain tissue at 37 °C (molar extinction coefficient of TBARS: 1.56 × 10^5^ M^−1^cm^−1^).

*Statistical Analysis:* All data were represented as Mean ± SEM and evaluated using a one-way ANOVA followed by a Tukey’s multiple comparison’s posttest. The X-rays of the western blot were scanned, compiled and the statistical analysis of relative densities was carried out using imageJ, GraphPad Prism5, and Adobe Photoshop. The densities of the proteins were expressed in arbitrary units (A.U.s) given as the Mean ± S.E.M. The “#” symbol denotes the significant difference from normal control to treated groups, while “*” denoted a significant difference from the scopolamine treated mice group, respectively; *, # *p* < 0.05, **, ## *p* < 0.01, ***, ### *p* < 0.001.

## 3. Results

**Figure 2:**
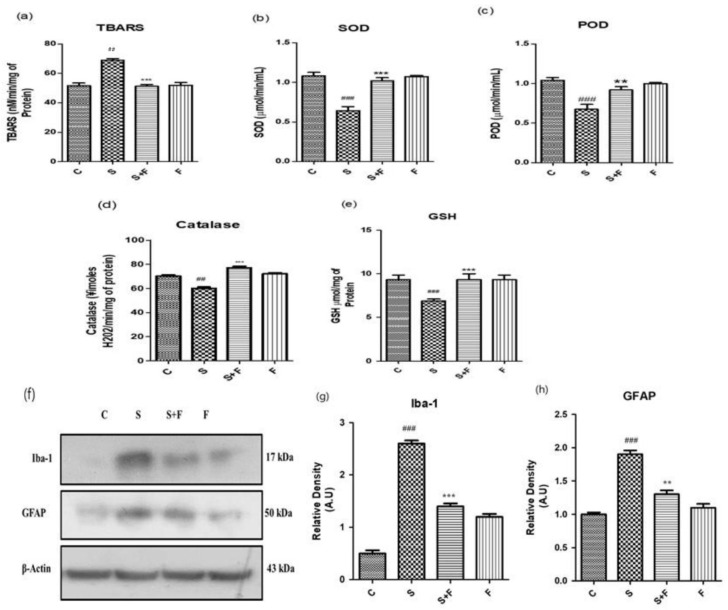
Friedelin abolished scopolamine-induced oxidative stress and glial cell activation in adult mice brain. Shown are the different antioxidant enzyme assays (**a**) Lipid peroxidase, TBARS (**b**) Superoxide dismutase, SOD (**c**) peroxidase enzyme, POD (**d**) Catalase enzyme, CAT and (**e**) Glutathione, GSH performed with brain homogenates of experimental mice treated either with scopolamine alone or in combination with friedelin, respectively. (**f**) Shown are western blot bands of glial cells along with respective histograms at (**g**,**h**). The treatment details have already been given in the materials and methods section. The results are stated as Mean ± S.E.M (*n* = 5). Significance of control vs. scopolamine is expressed as *#*, while * denotes scopolamine Vs scopolamine + friedelin. Significance: **, *## p* < 0.01 and ***, *### p* < 0.001.

*Friedelin Reduced Scopolamine-induced Oxidative Stress Mediated Glial Cells Activation in Mice:* Scopolamine was administered to the adult albino mice for three weeks once daily. After completion of the first week, friedelin was administered for the final two weeks and the brain homogenates of all mice were tested for the estimation of diverse antioxidant enzymes, i.e., SOD, POD, GSH, CAT and TBARS to establish the extent of oxidative stress induced by scopolamine. The results showed that scopolamine administration significantly amplified the oxidative stress in the mice brain by decreasing the SOD, POD, GSH, CAT levels and inducing TBARS activity in the adult mice brain. On the contrary, the administration of friedelin significantly increased the enzymatic activity of SOD, POD, GSH and CAT while TBARS activity declined, as shown in Figure 2a–e.

The oxidative stress characterized by an upsurge in reactive oxygen species and the down regulation of the counterfeit antioxidant system in the body. One of the main reasons for progressive neurodegenerative diseases, specifically AD, is the state of chronic oxidative stress and the dysregulation of the inflammatory responses [30,31]. The glial cells’ activation is considered as an important consequence in response to oxidative stress conditions [32]. Normally, glial cells including astrocytes and microglia are involved in the maintenance of homeostasis in the neuronal environment by regulating the movement of ions or neurotransmitters through the axons and synapses [33]. Scopolamine can lead to the activation of glial cells as well as impaired cellular antioxidant defence mechanisms, resulting in a progressed disease condition. In the current study, friedelin significantly inhibited the protein expressions of glial cells, which confirms its role as a counterfeit moiety for the management of oxidative stress, as shown in Figure 2f–h.

**Figure 3:**
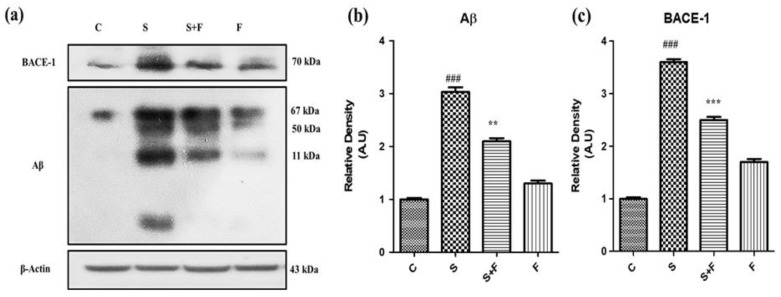
Friedelin abolished the amyloidogenic pathway of Aβ production against scopolamine. The immune blot analysis of markers of amyloidogenic pathway i.e., BACE-1 and Aβ of control vs. scopolamine and combination treatment of scopolamine and friedelin is shown in (**a**), while the respective histograms are placed at (**b**,**c**). The results were expressed in arbitrary unit (A.U) and were determined using ImageJ software and histogram indicate mean A.U ± SEM. Significance of control vs. scopolamine is expressed as *#*, while * denotes scopolamine vs. scopolamine + friedelin. ** *p* < 0.01 and ***, ### *p* < 0.001.

*Friedelin abolished the amyloidogenic pathway of Aβ production against Scopolamine:* Research findings have confirmed the participatory role of scopolamine for producing Aβ in animal brain [7]. In this study we have investigated the level of expression of Aβ protein and BACE-1 in all groups of experimental animals using the western blotting technique. The immuno-blot results clearly demonstrate that scopolamine administration significantly triggered β-secretase activity to cut the amyloid precursor protein at the sites of formation of toxic Aβ fragments, leading to Aβ production via the amyloidogenic pathway. The Aβ and BACE-1 levels are shown in the figure with respective treatment (Figure 3a). Friedelin being a neuroprotective agent significantly inhibited the BACE-1, accompanied by less production of Aβ in the brains of experimental mice, as presented in Figure 3a–c.

**Figure 4:**
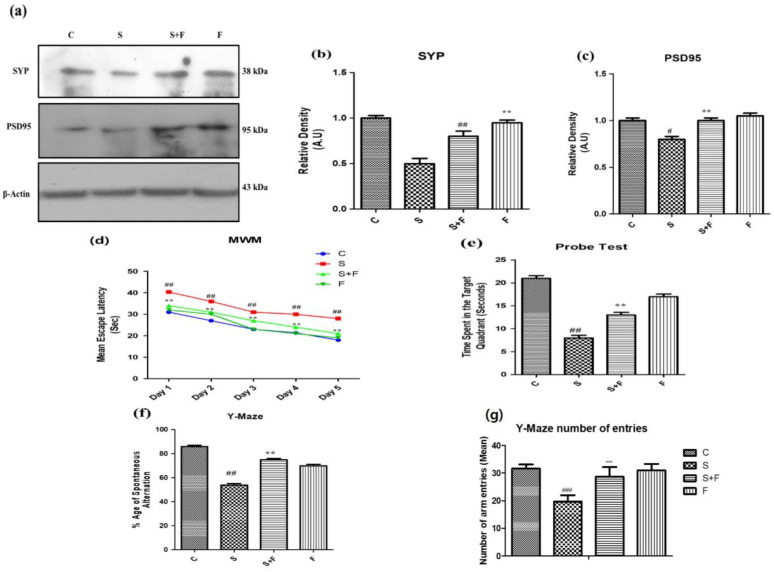
Friedelin improved pre and post synapse and memory dysfunction against scopolamine in mice. The up-regulation of SYP and PSD95 in response to administration of friedelin along with scopolamine in contrast to the control and scopolamine group is shown at (**a**), along with the histogram plot of respective relative densities (**b**,**c**). The results were expressed in arbitrary unit (A.U) and were determined using Image J software and histogram indicates mean in A.U ± SEM. While results of behavioral tests are given as (**d**) mean escape latency in MWM (**e**) probe test, (**f**) %age spontaneous alteration in Y-Maze test and (**g**) Y-Maze number of arm entries. Significance of control vs. scopolamine is expressed as *#*, while * denotes scopolamine vs. scopolamine + friedelin. Significance: *# p* < 0.05, **, *## p* < 0.01 and ***, *### p* < 0.001.

*Friedelin improved synapse (pre and post) reverse memory dysfunction against scopolamine in mice:* Scopolamine administration can significantly affect both pre- and post- neuronal synapse [34]. Neuronal pre- and post-synaptic proteins including synaptophysin (SYP) and PSD95 are of prime importance for neuronal communication. Their expression was evaluated through western blotting. The western blot results showed that scopolamine inhibited pre- and post-synapse protein expression in brains of male adult mice (Figure 4a), while friedelin administration along with scopolamine significantly protected the neuronal plasticity at synaptic junction. The protein expression level of both pre- and post-synapse was observed to be upsurged with the administration of friedelin.

Scopolamine is a chemical agent that affects the memory and behavior of the experimental animals [35]. In the current study the mice were injected with scopolamine and scopolamine + friedelin along with control and friedelin alone. The treated animals were subjected to two well-known behavioral tasks, the Morris water maze (MWM) and the Y-maze. In the MWM test the mice trained for two days (twice a day), then after giving them one day of rest, the data was collected for five (5) consecutive days. Their mean escape latencies were measured on a daily basis to reach the platform in one minute. On the first day, the mean escape latency of control animals was low, while scopolamine- treated mice have shown significantly higher mean escape latency (Figure 4d). In contrast, the group which received scopolamine along with friedelin showed significantly lesser mean escape latencies to reach the target platform. This trend was continued in all experimental groups, including the control animals and animals treated with only friedelin, which showed progressively less mean escape latencies. Although the scopolamine treated animals exhibited little improvement from day one to day five, overall their mean escape latencies were higher than that of the control animals. Moreover, in the probe test, after the removal of the hidden platform, the control and friedelin treated animals spent more time in the target quadrant, as given in the Figure 4e. In contrast, the scopolamine treated animals spent less time in the target quadrant. Interestingly, the mice which received friedelin in combination with scopolamine spent more time in the target quadrant, as presented in Figure 4f.

In the Y-maze behavioral task, the locomotor activity in terms of total arm entries made by mice in each group and the percentage of spontaneous alternations were observed to serve as an indicator of performance of spatial memory. The mice in the control group and the scopolamine treated groups showed the highest and lowest levels of locomotion, respectively. Friedelin treatment alone and in combination with scopolamine significantly enhanced the locomotion of mice in the respective groups. The percentage of spontaneous alternation of control animals was higher in comparison to scopolamine treated group of mice, while the group administered with friedelin + scopolamine displayed a higher percentage of spontaneous alternation, but overall it was less than the control animals, as shown in Figure 4f.

Friedelin specifically inhibited phosphorylated JNK activation to abrogate scopolamine-induced neuroinflammation and neurodegeneration in mice: In the current study scopolamine administration caused up-regulation of phosphorylated JNK in adult mice brain. The administration of scopolamine daily for 21 days resulted in oxidative stress induced neuroinflammation and neurodegeneration, attributed to phosphorylation of JNK, resulting in its activation. Administration of friedelin specifically inhibited phosphorylated JNK according to the western blot results. This inhibition is accompanied by reduction in oxidative stress, neuroinflammation, neurodegeneration, neuronal synapse improvement along with memory and cognition reversal. So all these beneficial effects of friedelin can be attributed to its capability to inhibit the phosphorylated JNK.

**Figure 5:**
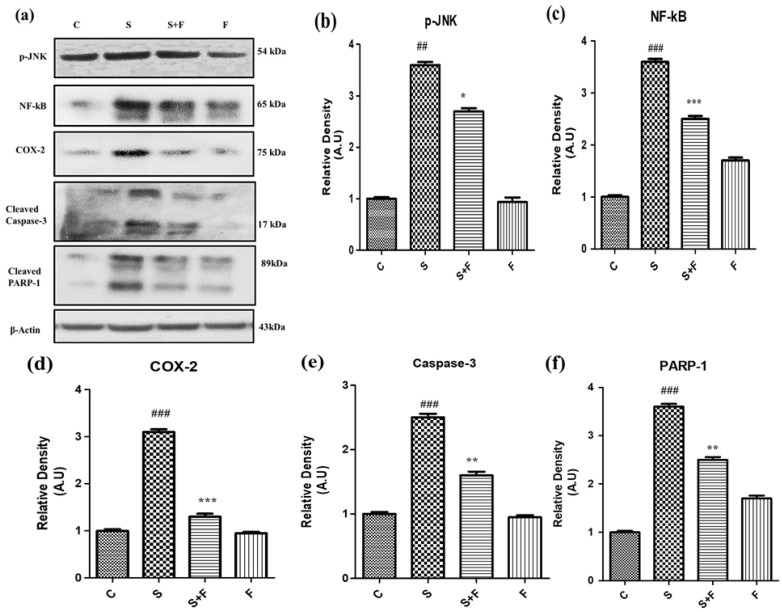
Friedelin inhibited p-JNK and abrogated scopolamine-induced neuroinflammation and neurodegeneration in mice. The immune-blot analysis of neuro-inflammatory markers (**a**) p-JNK, NF-kB, COX-2, caspase-3 and PARP-1 along with histograms of respective relative densities is shown at (**b**)p-JNK (**c**) NF-kB, (**d**) COX-2, (**e**) Caspase-3, (**f**) PARP-1 and β-Actin was used as the loading control. The results were expressed in arbitrary units (A.U) and were determined using Image J software and histogram indicate mean A.U ± SEM. Significance of control vs. scopolamine is expressed as #, while * denotes scopolamine vs. scopolamine + friedelin. * *p* < 0.05, **, *## p* < 0.01 and ***, *### p* < 0.001.

Scopolamine especially induced neuro-inflammatory markers such as NF-κB, by inducing its activation and translocation into the nucleus accompanied by the activation of COX-II (Figure 5a–d). Moreover this neuroinflammation was followed by neurodegeneration as scopolamine significantly triggered neuro-apoptotic markers such as caspase-3 (apoptotic executioner) leading to the damage of nucleus i.e., an increased expression of PARP-1 proteins to cause neuronal cell death as shown in Figure 5. Interestingly the administration of friedelin to the adult albino mice not only significantly abolished NF-κB and COX-II but also attenuated the neurodegenerative hallmarks comprising of caspase-3 and PARP-1 (Figure 5e,f).

**Figure 6:**
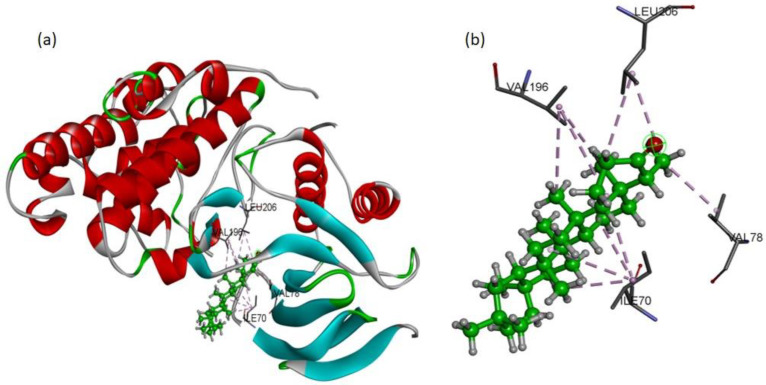
Docking pose of friedelin with p-JNK ligand. (**a**) Friedelin’s binding mode as inhibitor of JNK; the ligand is presented in a bright green color (**b**) 3D binding mode of friedelin with active site residues showing alkyl hydrophobic interactions (shown by purple dashed lines).

To validate the western blot results, the molecular docking studies were directed to elucidate the inhibitory potential of friedelin on p-JNK. Among the several conformations, the most promising docking pose was detected inside the p-JNK binding pocket with appropriate orientation. The p-JNK binding site is comprised of both the hydrophilic and hydrophobic amino acids. The hydrophobic amino acids include Ile70, Val78, Ala91, Ile124, Met146, and 149, Ala151, Val196, and Leu206, while the hydrophilic amino acids include Gly71, Ser72, Gly73, Gln75, Gly76, Lys93, Glu147, Asp150, Cys154, Gln155, 158, Ser193, Asn194, and 255. The analysis of binding modes of the most favorite docked conformation exposed that friedelin interacted well over the binding cavity with hydrophobic amino acids (Ile70, Val78, Val196, and Leu206) through multiple alkyl interactions with minimum distance of 3.60 Å (shown by purple dashed lines). Figure 6 depicts the 3D interactions of the most favorable conformation. Thus, friedelin showed efficient binding in terms of good binding affinity (−7.9 Kcal/mol). Such a lower value indicated the good fitness of the compound in the binding pocket of JNK protein and a stable friedelin-protein interaction. These theoretical results confirmed that the friedelin showed much better activity by making numerous interactions with JNK protein key residues.

**Figure 7:**
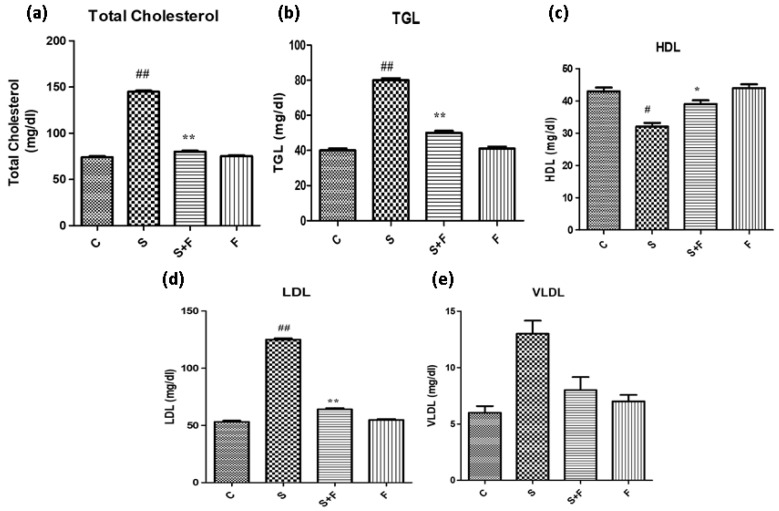
Friedelin reduced blood cholesterol and triglyceride abundance in adult mice. (**a**) TC levels, (**b**) TG levels, (**c**) HDL levels, (**d**) LDL and (**e**) VLDL levels are given in histogram. The results are expressed as mean ± SEM employing one-way ANOVA followed by Tukey’s multiple comparison post-test. Significance of control vs. scopolamine is expressed as #, while * denotes scopolamine vs. scopolamine + friedelin/glutinol. Significance: ns = non-significant *, *# p* < 0.05, **, *## p* < 0.01 (*n* = 5).

*Friedelin reduced blood cholesterol and triglycerides abundance in adult mice:* Scopolamine administration caused an increase in the cholesterol levels of adult mice. It is in accordance with a previous study in which scopolamine led to increased total cholesterol and triglycerides in experimental animals [36]. The results indicate that scopolamine significantly enhanced total cholesterol, TGL, LDL and VLDL, accompanied by the reduction in HDL in the serum of only scopolamine treated animals as shown in Figure 7. In contrast, the administration of friedelin significantly improved the blood parameters by lowering the total cholesterol, TGL, HDL, LDL and enhancing VLDL in the serum of the experimental animals, as shown in Figure 7.

## 4. Discussion

In the present findings, friedelin has shown neuroprotective effects against scopolamine-induced multiple neuropathological conditions of Alzheimer’s disease. Friedelin, being a triterpenoid isolated from *Quercus dilatata*, displayed unique and potent characteristics in treating the neuronal communication gap attributed to the administration of scopolamine to adult mice. Additionally, it minimized not only the oxidative stress but also the neuroinflammation and neurodegeneration mediated memory dysfunction via phosphorylated JNK inhibition. Interestingly, it also significantly reduced the cholesterol and improved the lipid profile of the mice challenged with scopolamine.

During the study we observed that friedelin had shown anti-oxidant capabilities, as it caused the up regulation of the levels of endogenous antioxidant enzymes i.e., SOD, POD, CAT, GSH; enzymes which are responsible for the decrease of oxidative stress. It also diminished the overexpressed TBARS. In the case of reduced expressions of antioxidant enzymes, oxidative stress may lead to neuronal damage and neuronal apoptosis [30]. A similar work was also reported on in which a nsuccinamide derivative significantly ameliorated scopolamine-induced antioxidant enzymes inhibition, which led to the recovery of the memory and relevant complications [33]. Yet another study reported the correlation of glial cells activation with the disease pathology and suggested that the molecules targeting the inhibition of glial cells and astrocytes can be a potential candidate for treatment of Alzheimer’s disease [37]. Strong evidence suggests that increased cholesterol level is a potential risk factor in the progression of Alzheimer’s disease complications. Increased cholesterol levels in neuronal membranes can facilitate Aβ production and its aggregation [38]. A recent study reported that LPS, a pro-inflammatory factor, significantly increased intracellular cholesterol accumulation by up-regulating the expression of NF-κB signalling by knocking-down overexpressed IKKα and TAB3. Interestingly, the increased levels of cholesterol contrariwise exerted an amplified pro-inflammatory effects by activating the NF-κB signalling pathway [39], as also confirmed by our results. These results may be suggestive of the correlation of serum cholesterol in correspondence with the overexpression of NF-κB and its downwards signalling pathways.

The up-regulation of neuroinflammatory and neurodegenerative biomarkers is another hallmark of oxidative damage. Friedelin has shown anti-inflammatory and anti-neuroapoptotic effects by lowering these markers such as NF-κB. It also inhibited the neuro-inflammatory process by inhibiting p-JNK, which is activated in case of increased oxidative stress conditions [40,41]. The inhibition of JNK phosphorylation was also confirmed through molecular docking and western blotting analyses. The p-JNK is further responsible for the decreased level of pre-and post-synapse proteins; hence memory dysfunction induced in scopolamine-treated mice. Our results of MWM and Y-Maze clearly indicates that friedelin has the ability to improve both long and short term memory deficits in mice. The performance of the friedelin treated mice in MWM was far better than mice treated with scopolamine alone because they have shown reduced mean escape latencies on a regular interval throughout the testing period and displayed enhanced spatial memory, accompanied by more time spent in the target quadrant in the probe test. Although their performance was better than the scopolamine-treated mice, they still were less efficient than the control group mice, which suggests that these mice have partially recovered from the scopolamine-induced dysfunction in long-term memory. Furthermore, in a Y-Maze experiment, friedelin administered mice displayed a greater percentage of spontaneous alterations as compared to the mice that only received scopolamine.

The production of Aβ from amyloid precursor protein (APP) protein through the amyloidogenic pathway is another well-established characteristic of Alzheimer’s disease [42]. β-secretase (BACE-1) is a major secretase enzyme which causes the fragmentation of APP protein [43]. Interestingly, the current study demonstrated that triterpenoid friedelin efficiently inhibited β-secretase activity and abolished the production of Aβ. The reduction in Aβ production can be attributed to reduced serum cholesterol and triglyceride levels and enhanced antioxidant enzyme activity in friedelin-treated mice.

## 5. Conclusions

After summarizing the results, it can be suggested that scopolamine is neurotoxic, as it compels the normal brain to induce AD neuropathology in adult mice. Friedelin may be considered as a bioactive therapeutic agent that reverses all of the neuropathological symptoms induced by scopolamine. These results are of clinical importance because for the first time these findings are suggestive of a new investigational candidate as a potential lead for the treatment of AD by targeting multiple markers including those of amyloidogenic pathology.

## Figures and Tables

**Table 1:** Chromatogram, 3D graph and UV spectrum of the friedelin sample and the standard.

Standard	Sample
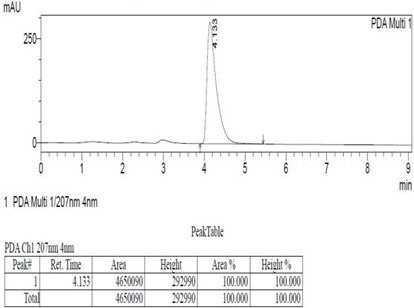	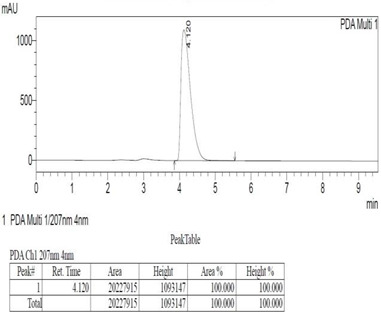
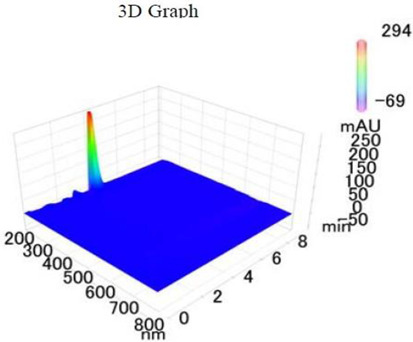	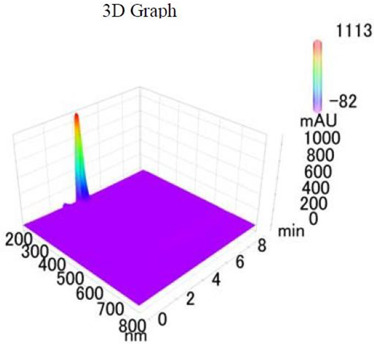
Spectrum 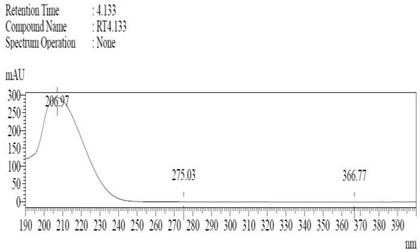	Spectrum 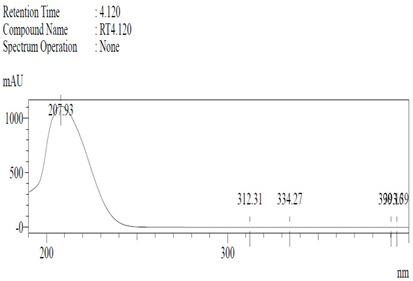

## Data Availability

Data will be available on request.
